# Inhaled NO at a crossroads in cardiac surgery: current need to improve mechanistic understanding, clinical trial design and scientific evidence

**DOI:** 10.3389/fcvm.2024.1374635

**Published:** 2024-04-05

**Authors:** Stefan Muenster, Iratxe Zarragoikoetxea, Andrea Moscatelli, Joan Balcells, Philippe Gaudard, Philippe Pouard, Nandor Marczin, Stefan P. Janssens

**Affiliations:** ^1^ Department of Anesthesiology and Intensive Care Medicine, University Hospital Bonn, Bonn, Germany; ^2^ Department of Anesthesiology and Intensive Care Medicine, Hospital Universitari I Politècnic Fe, Valencia, Spain; ^3^ Neonatal and Pediatric Intensive Care Unit, Emergency Department, IRCCS Istituto Giannina Gaslini, Genova, Italy; ^4^ Pediatric Intensive Care Unit, Vall d’Hebron Barcelona Campus Hospitalari, Universitari Vall d'Hebron, Barcelona, Spain; ^5^ Department of Anesthesiology and Critical Care Medicine Arnaud de Villeneuve, CHU Montpellier, University of Montpellier, PhyMedExp, INSERM, CNRS, Montpellier, France; ^6^ Department of Anesthesiology and Critical Care, Assistance Publique-Hopitaux de Paris, Hopital Necker-Enfants Malades, Paris, France; ^7^ Department of Surgery and Cancer, Imperial College, London, United Kingdom; ^8^ Cardiac Intensive Care, Department of Cardiovascular Diseases, University Hospital Leuven, Leuven, Belgium

**Keywords:** inhaled nitric oxide, pulmonary hypertension, pediatric cardiac surgery, adult cardiac surgery, right heart failure

## Abstract

Inhaled nitric oxide (NO) has been used in pediatric and adult perioperative cardiac intensive care for over three decades. NO is a cellular signaling molecule that induces smooth muscle relaxation in the mammalian vasculature. Inhaled NO has the unique ability to exert its vasodilatory effects in the pulmonary vasculature without any hypotensive side-effects in the systemic circulation. In patients undergoing cardiac surgery, NO has been reported in numerous studies to exert beneficial effects on acutely lowering pulmonary artery pressure and reversing right ventricular dysfunction and/or failure. Yet, various investigations failed to demonstrate significant differences in long-term clinical outcomes. The authors, serving as an advisory board of international experts in the field of inhaled NO within pediatric and adult cardiac surgery, will discuss how the existing scientific evidence can be further improved. We will summarize the basic mechanisms underlying the clinical applications of inhaled NO and how this translates into the mandate for inhaled NO in cardiac surgery. We will move on to the popular use of inhaled NO and will talk about the evidence base of the use of this selective pulmonary vasodilator. This review will elucidate what kind of clinical and biological barriers and gaps in knowledge need to be solved and how this has impacted in the development of clinical trials. The authors will elaborate on how the optimization of inhaled NO therapy, the development of biomarkers to identify the target population and the definition of response can improve the design of future large clinical trials. We will explain why it is mandatory to gain an international consensus for the state of the art of NO therapy far beyond this expert advisory board by including the different major players in the field, such as the different medical societies and the pharma industry to improve our understanding of the real-life effects of inhaled NO in large scale observational studies. The design for future innovative randomized controlled trials on inhaled NO therapy in cardiac surgery, adequately powered and based on enhanced biological phenotyping, will be crucial to eventually provide scientific evidence of its clinical efficacy beyond its beneficial hemodynamic properties.

## 1 Introduction

**Figure 1:**
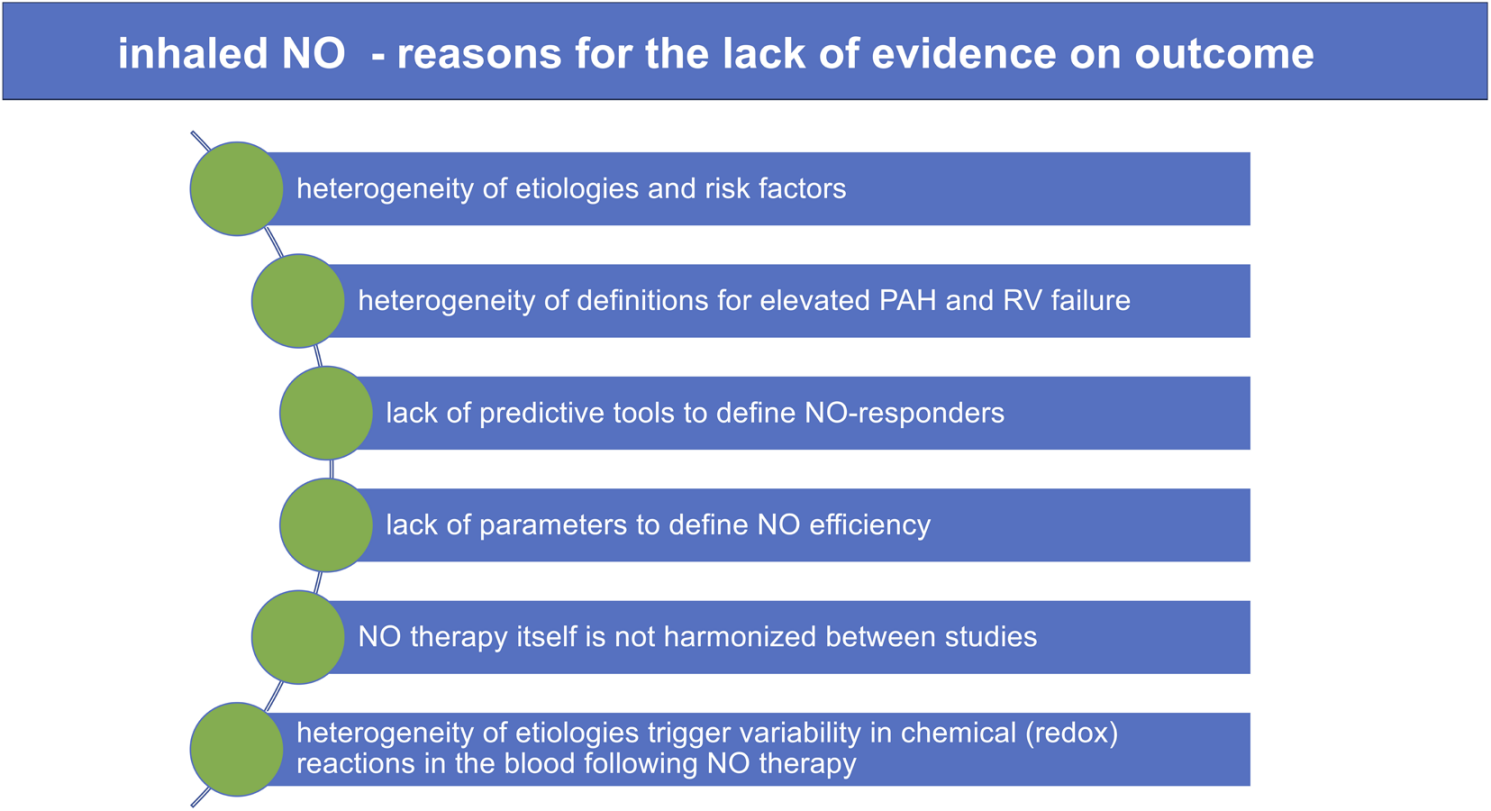
Inhaled NO: reasons for the lack of evidence on outcome. NO, nitric oxide; PAH, pulmonary arterial hypertension; RV failure, right heart failure.

The use of inhaled nitric oxide (NO) in perioperative cardiac surgery has its origin in the early studies of Frostell and colleagues in 1991, where these authors demonstrated that breathing low concentrations of gaseous NO lowered pulmonary artery pressure in awake lambs with experimental pulmonary hypertension (PH) (1). Ever since multiple researchers studied inhaled NO and its various cardiopulmonary effects in animals and humans—efforts that eventually led to the approval of inhaled NO by the U.S. Drug and Food Administration in 1999 and the European Medicine Evaluation Agency and European Commission in 2001 (2). Although its beneficial effects on acutely lowering pulmonary artery pressure and reversing right ventricular (RV) dysfunction and/or failure have been reported in numerous studies, these investigations failed to demonstrate significant differences in survival or other clinical outcome parameters (Figure 1) (3, 4).

The authors have collaborated as an expert advisory board in the field of inhaled NO in pediatric and adult cardiac surgery to demonstrate in this review how the current scientific evidence for the use of inhaled NO in perioperative cardiac surgery can be further improved. We first provide a brief overview of the mechanism of action of inhaled NO and then list the major postoperative morbidities associated with cardiac surgery that could be treated with inhaled NO according to the above mechanisms. We then provide a brief analytic overview of the current state of research on inhaled NO, discuss which approaches have failed, what has been shown to be effective, and which approaches might have provided scientific evidence for improved outcomes with inhaled NO, yet failed to do so because of inadequate study design. We will talk about how biomarkers may help to identify the target population, how they could improve and optimize inhaled NO therapy, and help to guide more robust and informative randomized controlled trials in the future.

## 2 Basic mechanisms of NO underpinning clinical applications

### 2.1 NO and its cellular signaling pathway

Endogenous NO is an important cellular signaling molecule with multiple biological actions (2, 5) and is synthetized from L-arginine and oxygen by a family of three isoenzymes, called NO synthases (NOSs). Downstream NO signaling in the cardiovascular system involves two major biochemical mechanisms: (1) activation of the heme-containing protein guanylate cyclase where NO binds to the reduced heme (Fe^2+^) with formation of the second messenger cGMP and activation of cGMP-dependent protein kinases (PKG) and (2) nitrosylation of various low molecular compounds and proteins involved e.g., in cardiac contractility through direct radical interaction with sulfhydryl moieties and direct effects on circulating cells.

During normal cardiovascular homeostasis, the endothelial NOS isoform is critically important in modulating vasomotion of resistance vessels and maintaining a low pulmonary vascular tone through a cGMP-mediated NO effect resulting in vascular smooth muscle cell relaxation (6). Recent findings of decreased iNOS expression and deficient NO bioavailability in a rat model of hypoxia-induced PH suggest that the inducible NOS (iNOS) isoenzyme may also significantly impact on the development of PH (7). By successfully restoring iNOS expression using Adeno-Associated Virus (AAV)-mediated gene transfer, the subsequently increased NO production significantly reduced pulmonary artery pressure and improved RV function in treated compared to control rats.

With respect to cGMP-independent signaling, nitrosylation reactions of inhaled NO with proteins involved in cardiac contractility, such as L-type calcium channels and the ryanodine receptor, may modulate cardiac contractility and RV function in patients undergoing cardiac surgery via altered calcium cycling. Alternatively, NO can modulate immune cell function, reductions in pro-oxidant angiotensin II (Ang II) signaling, sympathetic nerve activity, and modulation of mitochondrial function, all of which may dose-dependently contribute to context-specific clinical effects.

### 2.2 Inhaled NO as a selective pulmonary vasodilator

**Figure 2:**
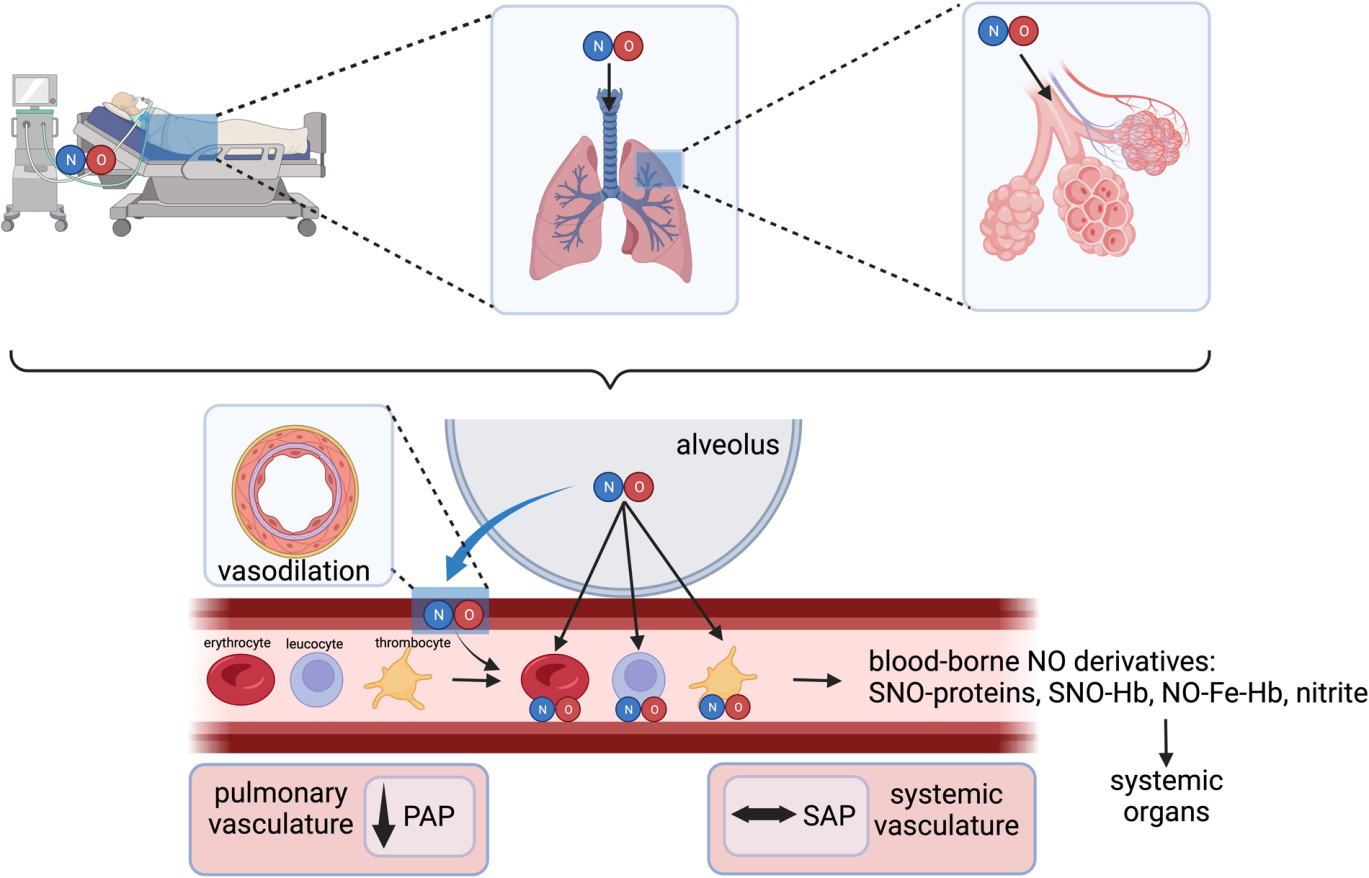
Inhaled NO as a selective pulmonary vasodilator and interactions with various blood components. NO, nitric oxide; PAP, pulmonary arterial pressure; SAP, systemic arterial pressure; SNO-Hb, S-nitrosohemoglobin; NO-Fe-Hb, ferrous nitrosyl-hemoglobin. Created with BioRender.com.

In cardiac surgery, inhaled NO has been widely used as a selective pulmonary vasodilator to rapidly treat acute PH, RV dysfunction, and/or failure (3). Gaseous NO is distributed only to ventilated lung regions and exerts the unique ability to selectively induce smooth muscle relaxation in the pulmonary vasculature in these regions (Figure 2) (5, 8). Thus, inhaled NO reduces intrapulmonary shunt and improves oxygenation by diverting pulmonary blood flow to the ventilated areas. In pediatrics, the reduction in pulmonary pressures also improves extrapulmonary right to left shunt (8, 9). NO crosses the alveolo-capillary membrane, and in the presence of oxygenated hemoglobin (Hb), NO is rapidly metabolized to nitrate with the formation of met-Hb—a mechanism that limits its action mainly to the pulmonary vasculature (10).

### 2.3 Effects of inhaled NO beyond selective pulmonary vasodilation

In addition to the well-recognized cGMP-dependent vasodilatory effects of inhaled NO in the lungs, several cGMP-independent effects have been well-recognized following exposure to NO gas inhalation. These include the formation of S-nitrosohemoglobin (SNO-hemoglobin) and chemical redox reactions with the formation of nitrate (NO^3−^) and nitrite (NO^2−^) (11). Nitrite serves as a stable vascular reserve of NO, from which it can be generated at distant sites through a reductive pathway facilitated by several heme-containing and molybdenum-cofactor proteins. This process is enhanced during physiological and pathological hypoxia and in disease states involving ischemia-reperfusion injury, such as during cardiopulmonary bypass (CPB) (12, 13).

Finally, inhaled NO may directly affect activated neutrophils and platelets as they pass through the lungs and mitigate their secretory, immune- and thrombosis-related properties, resulting in the de-activation of these blood components. These anti-inflammatory and antiplatelet properties of inhaled NO may target key pathogenic mechanisms in high-risk cardiovascular patients that result in myocardial and kidney dysfunction and injury.

These basic considerations have led to great enthusiasm for NO-based therapies in clinical conditions, where selective pulmonary vasodilation and anti-inflammatory effects are considered beneficial, as is notoriously the case in cardiac surgery.

## 3 The mandate of inhaled NO for cardiac surgery

**Figure 3:**
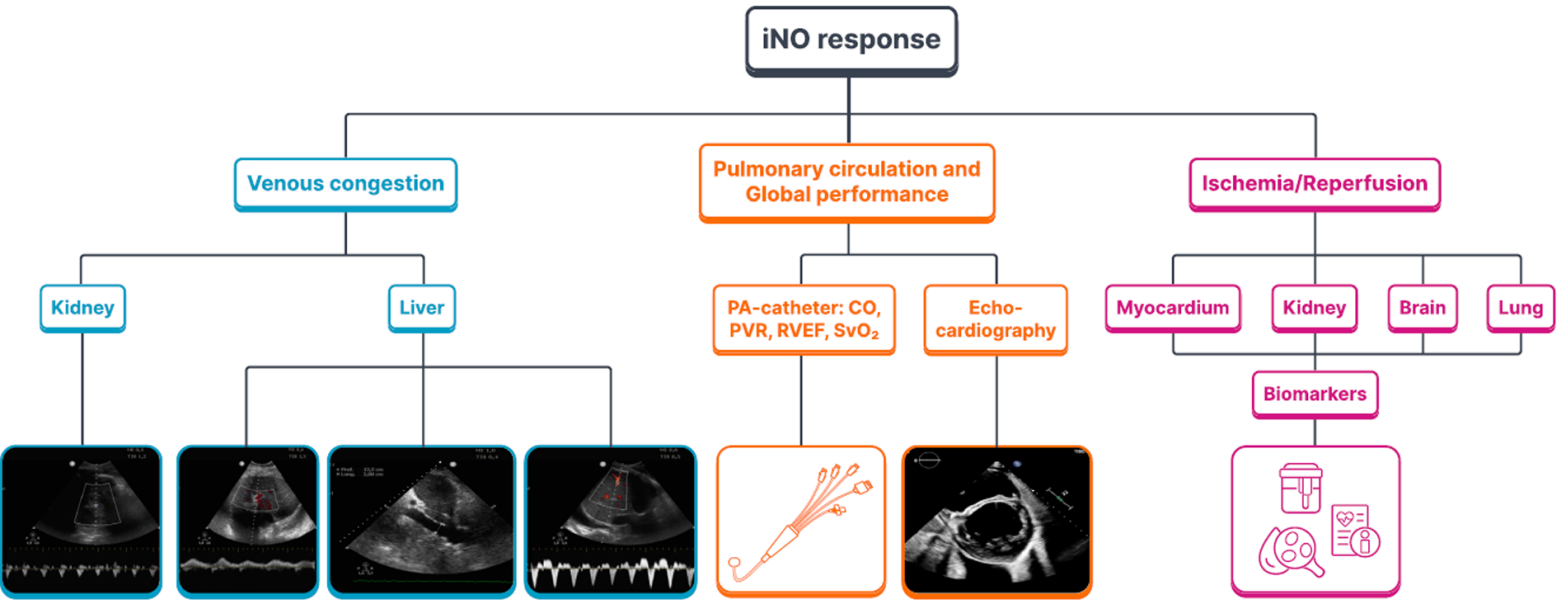
The mandate of inhaled NO for cardiac surgery: inhaled NO response to venous congestion, the pulmonary circulation and to the ischemia/reperfusion injury. iNO, inhaled nitric oxide; PA-catheter, pulmonary artery catheter; CO, cardiac output; PVR, pulmonary vascular resistance; RVEF, right ventricular ejection fraction; SvO2, venous oxygen saturation.

Inhaled NO therapy may improve the outcome of a cardiac surgery patient by targeting two critical events. First, preexisting PH and increased pulmonary vascular resistance (PVR) are complex risk factors that influence postoperative morbidity and mortality predominantly by an effect on RV dysfunction and consequent circulatory failure (14) (Figure 3). By selective pulmonary vasodilation, inhaled NO may improve pulmonary (and systemic) hemodynamics and prevent low cardiac output syndrome. Second, as NO appears to be a master regulator of local myocardial ischemia and reperfusion injury and a modulator of pulmonary and systemic inflammatory responses associated with cardiac surgery, inhaled NO may modulate inflammatory outcomes such as myocardial damage and postoperative myocardial, pulmonary, or kidney dysfunction and injury (15) (Figure 3). Importantly, these hemodynamic and immunomodulatory events are interrelated with systemic inflammation, worsening RV performance, and postoperative PVR and RV dysfunction augmenting the inflammatory response through venous congestion, bacterial translocation, and subsequently multiorgan dysfunction syndrome (MODS).

### 3.1 Pulmonary hypertension

Pre-existing and acute post-CPB pulmonary hypertension is a recognized risk factor for complications after cardiac surgery, and the presence of PH is a significant component of EuroSCORE (16). While there is profound scientific evidence of pre-existing PH, there is only limited research and data on the global incidence and impact of PH after CPB and cardiac surgery.

In fact, pre-existing PH is a relatively common complication of chronic heart disease (CHD), both in acquired heart disease in adults (valvular, myocardial, and pulmonary lesions) or CHD in the pediatric population. In adult cardiac surgery, patients with left ventricular failure and left valvular disease exhibit a not fully elucidated PVR increase. It might be partly attributable to the impaired release of endothelial NO in pulmonary resistance vessels in response to altered endothelial shear stress and elevated pressures in the left heart chambers, resulting in elevated pressures in the pulmonary vascular system (17). This chronic condition can induce remodeling of the pulmonary vessels, leading to pressure overload and postcapillary or both pre- and post-capillary PH, respectively (18).

In children, pre-existing PH is mainly observed with congenital heart lesions with either long-standing pulmonary overcirculation (e.g., ventricular septal defect, atrioventricular canal, transposition of the great arteries, truncus arteriosus, aortopulmonary window) or with obstructive lesions (e.g., totally anomalous pulmonary venous return, Shone's syndrome) (19). Beyond the hemodynamic forces, endothelial dysregulation of the pulmonary vasculature also seems to be a contributing factor to postoperative PH, including an imbalance of vasodilator and vasoconstrictor mechanisms in part due to deficiency of NO pathways and an increased expression of vasoconstrictors such as endothelin-1 and big-endothelin (20).

Postoperative PH can be aggravated by ischemia and subsequent endothelial injury of pulmonary resistance vessels, a condition that finally leads to the release of proinflammatory cytokines. All these deleterious effects lead to reduced endothelial NO bioavailability—a phenomenon that has been observed during CPB surgery (21, 22).

The “2022 European Society of Cardiology (ESC)/European Respiratory Society (ERS) Guidelines for the diagnosis and treatment of pulmonary hypertension” provided a new definition (mPAP > 20 mmHg at rest) and classification of PH (23). Unlike PH associated with adult congenital heart disease included in group 1 of the PH clinical classification, PH associated with left heart disease constitutes group 2, including heart failure with reduced ejection fraction (HFrEF), heart failure with preserved ejection fraction (HFpEF), valvular heart disease and congenital/acquired cardiovascular conditions leading to post-capillary PH. Group 2 PH is characterized by a pulmonary artery wedge pressure >15 mmHg and divided into two categories: isolated post-capillary PH defined by PVR ≤ 2 Wood Unit (WU) and combined post- and pre-capillary PH defined by PVR > 2 WU. A PVR > 5 WU may indicate a severe pre-capillary component of the PH and carries the worst prognosis.

In summary, pre-existing and acute post-CPB pulmonary hypertension represent important challenges for the management of cardiac surgery patients and are the principal target for perioperative pulmonary vasodilatory treatment to treat or avoid a PH crisis after CPB and its detrimental consequences on RV function in high-risk cardiac surgery, transplantation, and left ventricular assist device (LVAD) implantation. Thus, the main promise and mandate of inhaled NO therapy in cardiac surgery is to preserve cardiac output and to manage low cardiac output-related postoperative morbidity and mortality.

### 3.2 Impact of NO on ischemia/reperfusion injury, systemic inflammation, and organ dysfunction

Most open-heart surgeries require a motionless and bloodless heart that is achieved by aortic cross-clamping, thereby inducing global myocardial ischemia and necessitating adequate myocardial protection through cardioplegia. However, despite various cardioplegic strategies, this protection is incomplete, and many biological studies imply a degree of myocardial ischemia/reperfusion injury affecting both the right and left ventricles (24). Similarly, the technical conduct of CPB also confers pulmonary ischemia by directing the pulmonary blood flow away from the lung vasculature. In addition, the most common non-pulsatile flow also reduces regional blood flow to systemic organs such as the kidney, liver, and gut and impacts cerebral circulation (25, 26). Hemolysis and cell-free hemoglobin subsequently confer additional systemic vasoconstriction by scavenging endogenous endothelial-derived NO (27).

Finally, a systemic inflammatory response through monocyte, neutrophil, and platelet activation provides additional hits for multiple organ dysfunction. These ischemia, reperfusion, and superinduced systemic inflammatory events comprise additional direct myocardial mechanisms resulting in RV dysfunction. But at the same time, they have direct vascular effects resulting in dynamically elevated PVR and RV dysfunction, therefore worsening the ventricular-arterial coupling.

It is estimated that organ injury and dysfunction of the heart and kidneys affect over 40% of patients undergoing cardiac surgery and contribute to the majority of life-threatening complications of cardiac surgery and healthcare related costs. In the TITRE2 trial, acute kidney injury and low cardiac output occurred in 34% and 11% of all patients and contributed to 41% and 24% of all deaths, respectively (28). Our understanding of underlying mechanisms is poor, and there are no effective prevention strategies today. Of note, systematic reviews of nearly 1,000 randomized controlled trials (RCTs) of organ protection interventions administered in the immediate perioperative period have consistently shown disappointing results (29–32). In recent years, many of the previously widely accepted causes and pathogenic mechanisms of organ injury following cardiac surgery, including anemia, bleeding, transfusion, tissue hypoxia, and innate immune system activation, have been refuted (29–33). New approaches are required for clinical progress.

#### 3.2.1 Pulmonary dysfunction in cardiac surgery

Patients undergoing cardiac surgery are often at an increased risk of perioperative pulmonary dysfunction, a disease state of the lung that is characterized by PH, increased PVR, and various inflammatory processes. Moreover, underlying lung diseases, such as chronic obstructive pulmonary disease (COPD), pulmonary dysfunction secondary to cardiac disease (such as congestive heart failure and others), and the use of CPB during cardiac surgery are known risk factors for perioperative pulmonary dysfunction (3, 34). Benedetto and co-workers described three basic mechanisms that can cause increased PVR and subsequent pulmonary dysfunction (3). First, alveolar hypoxia triggers vasoconstriction of small, pre-capillary pulmonary arteries. Second, chronic hypoxia results in structural changes in pulmonary blood vessels, characterized by the thickening of the intimal and medial layers of the pulmonary vascular bed. Lastly, chronic lung diseases like emphysema can lead to loss of alveolar lung tissue, potentially causing a concomitant decrease in the number of pulmonary capillaries.

The use of CPB during cardiac surgery also negatively affects pulmonary function through an increased amount of cell-free hemoglobin (a potent scavenger of endothelial NO, resulting in reduced NO bioavailability in the pulmonary vasculature and increased PVR), and liberation of various cytokines and other inflammatory responses (27). In lung transplant surgery, the development of pulmonary dysfunction can be crucial for the onset of acute lung injury, perioperative lung edema or primary graft dysfunction (3).

#### 3.2.2 Acute kidney injury

Acute kidney injury (AKI) is a known and frequent complication in cardiac surgery. The reported incidence varies as the definition and the severity of AKI is inconsistent, but some studies showed an incidence of up to 80% (35). In addition, the onset of AKI is related to mortality, morbidity, and hospital length of stay (35). The mechanisms of AKI in cardiac surgery include renal vasoconstriction, hemolysis, ischemia, and inflammation.

Seemingly, the selective pulmonary vasodilator effects of inhaled NO would dictate that this therapy has no influence on organ dysfunction other than the lungs. However, in addition to the well-recognized cGMP-dependent vasodilatory effects of inhaled NO in the lungs, several cGMP-independent effects have been well recognized following NO gas inhalation in the blood compartment and at distant systemic sites. SNO-hemoglobin, and the NO oxidation products NO_2_^−^ and nitrate NO_3_^−^, have been proposed as a stable intravascular reservoir from which NO can be reconstituted at distant organ sites through a reduction step, which seems enhanced during ischemia/reperfusion injury and hypoxia.

Thus, inhaled NO therapy may influence these injurious mechanisms by virtue of its metabolic and cellular signaling effects and dynamically modulate vasodilation and tissue perfusion at distant organ levels. Hence, the potential reduction of myocardial, renal, cerebral, and pulmonary dysfunction by inhaled NO represents the second major mandate for pediatric and adult cardiac surgery.

## 4 Popular use of inhaled NO in cardiac surgery

Based on the above perceived mandate, inhaled NO therapy has been popular for licensed and off-label applications in pediatric and adult cardiac surgeries. Some registry data and various surveys on the use of inhaled NO in cardiac surgery reflect this apparent enthusiasm. Wong and co-workers retrospectively reviewed 40,000 pediatric cardiac surgical admissions in 2019 (36). Among patients with PH, 12% received inhaled NO, and nearly 2% of admissions received inhaled NO despite the absence of documented PH (36).

A recent survey initiated by the Board of the European Society of Anesthesiology and Intensive Care (ESAIC) and endorsed by the European Association of Cardiothoracic Anesthesiology and Intensive Care (EACTAIC) also indicated widespread use of inhaled NO in the cardiac surgery setting. Although suffering from a low response rate from members of these societies, the survey revealed that PH and RV failure represented the most frequent indications for inhaled NO use in modern clinical practice. For instance, 44% of respondents indicated routinely using inhaled NO in patients with RV dysfunction, in addition to 41% of respondents who used it only in exceptional cases of RV dysfunction. Prophylactic use of inhaled NO during heart and lung transplantation or LVAD implantation surgery was reported by 12%–34%. Importantly, 86% of respondents also routinely used alternative selective pulmonary vasodilators, including systemic phosphodiesterase type V inhibitors (64%), inhaled prostacyclins (43%), and systemic endothelin antagonists (36%). Inhaled phosphodiesterase type III inhibitors were used by 26% of respondents, and systemic sGC stimulators and activators were used by 9%.

A survey of higher risk cardiac surgery targeted a patient population with LVAD implantations and also independently documented high prophylactic or therapeutic use of inhaled NO as first line modality for RV dysfunction in both U.S. and European centers. Moreover, a survey into the management of lung transplantation suggested that most transplant centers used inhaled NO to treat PH associated with lung transplantation.

## 5 The evidence base for the use of inhaled NO in cardiac surgery

Numerous cohort studies, small RCT, and some real-life database studies support the physiological vasodilatory and organ protective benefits of inhaled NO. However, there is only limited evidence for translating this into meaningful clinical practice outcomes, reduction of postoperative morbidity and there is no evidence for mortality benefit. The fact that such benefit can be achieved with some alternative therapies and the high cost currently associated with inhaled NO applications, represents a crossroads for inhaled NO therapy. We face scientific, clinical, ethical and management obligations to improve this unique therapy and to uncover the barriers to improved outcomes. To do so, we need to clearly define and better understand the fundamental mechanisms of responsiveness of individual patients, or lack thereof, to inhaled NO-based therapy.

### 5.1 Effect on PH and RV function

A pairwise meta-analysis based on 10 RCTs investigating inhaled vasodilator agents in cardiac surgical patients with concomitant PH demonstrated an effect on hemodynamic and echocardiographic variables and a negligible increase in the length of ICU stay of about 16 h compared with placebo (*n* = 5 studies) (37). In contrast, a pairwise meta-analysis on inhaled NO in cardiac surgery without age restriction based on 18 RCTs, with parallel groups, demonstrated a minimal decrease in the duration of mechanical ventilation and intensive care unit (ICU) stay (38).

A recent systematic review of RCTs using pulmonary vasodilators with a Bayesian network meta-analysis selected twenty-eight studies randomizing 1,879 patients (39). Inhaled NO and inhaled prostacyclin were associated with a reduction in ventilation duration and ICU stay while none of the other interventions reached a statistically significant difference compared to usual care or placebo on secondary outcomes. However, only inhaled prostacyclin had a beneficial effect on mortality in this analysis.

### 5.2 Effect on myocardial injury

In patients with ST-elevation myocardial infarction, ischemia/reperfusion injury accounts for up to 50% of myocardial damage, and has been a target for various pharmacological and non-pharmacological “conditioning” strategies, although with inconsistent clinical translation (40). The therapeutic use of NO-donor compounds after myocardial infarction has been hindered by their systemic blood pressure lowering effects. In contrast, inhaled NO does not induce prohibitive systemic hypotension and has been reported to beneficially affect hemodynamics in right ventricular myocardial infarction and cardiogenic shock (41). Subsequent prospective randomized, double-blind, controlled trials with either inhaled NO or the NO-releasing vasodilator nitroprusside failed to reduce myocardial infarct size (42). Of note, inhaled NO significantly reduced infarct size and improved left ventricular (LV) functional recovery in 56% of patients that did not receive combinatorial treatment with intracoronary pharmacological NO-donors, which may chemically interact with inhaled NO to form toxic peroxynitrite intermediates (43). These preliminary observations emphasize the importance of the local and/or systemic inflammatory and redox milieu when administering NO via inhalation for organ protection.

### 5.3 Effect on pulmonary dysfunction

International guidelines support the use of inhaled NO after lung transplant to promote lung-allograft function by improving oxygenation and lowering PVR, thereby mitigating the development of severe (grade 3) primary graft dysfunction (44). Most studies examining the role of inhaled NO in preventing morbidity and mortality after orthotropic lung transplant focused on potential reductions in the incidence of ischemia/reperfusion injury as the determinant of clinical outcomes with variable results in terms of a reduction of incidence of ischemia/reperfusion injury (3, 45). Unfortunately, most of these trials are non-randomized, uncontrolled studies and suffer from small sample sizes. Larger multicenter trials should, therefore, provide information about future directions.

In contrast to NO's quintessential role in vascular endothelial homeostasis, its effect on airway epithelial cell function in different pathologic conditions is poorly understood. The fraction of exhaled nitric oxide (F_E_NO) is increased in asthma, chronic pulmonary obstructive disease (COPD), lung fibrosis, and cancer. Reactive oxygen species mediate further uncoupling of NO with subsequently increased peroxynitrite levels and greater bronchial epithelial barrier dysfunction (46). Hence, the administration of inhaled NO in patients with inflammatory lung disease undergoing cardiac surgery needs careful consideration and monitoring of potential pro-oxidative adverse effects.

### 5.4 Effect on AKI

A common and serious complication of cardiac surgical procedures that require prolonged CPB is AKI. The mechanisms which lead to the development of AKI are multifactorial. Recently, CPB-associated hemolysis and the subsequent release of ferrous oxy-hemoglobin into the circulation have been postulated to facilitate the development of intrarenal injurious oxidative reactions and to deplete vascular NO bioavailability via the deoxygenation reaction to form met-hemoglobin (27). In a phase IIb RCT in 244 young Chinese cardiac surgical patients with rheumatic fever undergoing multiple valve replacement, the inhalation of 80 ppm NO during CPB and for 24 h postoperatively showed promising signals on perioperative AKI (27). Whether or not inhaled NO will effectively prevent AKI in older patients with cardiovascular risk factors and dysfunctional endothelium, remains to be determined and is currently under investigation (47).

Of note, early studies in patients with acute respiratory distress syndrome (ARDS) found that prolonged use of inhaled NO may increase the risk of AKI, possibly by the formation of reactive nitrogen species (48–50). While the exact mechanism of NO-related renal toxicity in ARDS patients remains unclear, several scientific societies have limited recommendations for the treatment of ARDS patients with inhaled NO to patients who remain severely hypoxemic despite optimal mechanical ventilation and other rescue strategies (51–54).

### 5.5 Take home messages from clinical trials

The overall message of these trials is that the reproducible physiological benefit (on PH and some aspects of inflammation) by inhaled NO (and other pulmonary vasodilators) do not readily translate to meaningful and long-term clinical outcomes on morbidity and mortality, thereby providing only weak and inadequate evidence for routine use. Despite such reality, expert consensus and clinical practice guidelines recognize the perioperative management difficulties (in high-risk cardiac surgery) and the potential of inhaled NO, which resulted in practice-based recommendations for the use of inhaled NO in these circumstances (4, 55).

These developments leave the practicing clinician at a major dilemma and uncertainty of management of these high-risk patients. Nevertheless, inhaled NO continues to be implemented in standard protocols because of its recognized benefit in pathophysiology. However, the cost pressure and the similar benefit achievable with alternative therapies increasingly forces centers to abandon inhaled NO and redirect practice towards inhaled prostacyclin.

Thus, inhaled NO therapy for cardiac surgery has reached a crossroads where its future depends on further evidence that (1) inhaled NO therapy can be improved, (2) the fraction of non-responders can be reduced, (3) the magnitude of physiological response can be augmented and (4) translation to major clinical outcome can be demonstrated.

## 6 Clinical and biological barriers, and gaps in knowledge: considerations for future research

### 6.1 Phenotypes of inhaled NO responders and non-responders: role of vasoreactivity testing

Pulmonary vasoreactivity testing is an important tool for assessing the reversible component of PH and distinguishing between the inhaled NO responder and the non-responder phenotype. The recent ESC/ERS guidelines provide important insights into this field and represent an international consensus on what the clinical community considers a clinical target response (23). Such testing is recommended in patients with idiopathic, heritable pulmonary arterial hypertension or associated with drugs and toxins or in selected left heart disease patients (e.g., evaluation for heart transplantation, before systemic-to-pulmonary shunt or atrial-ventricular septal defect closure). The updated ESC/ERS 2022 guidelines define a positive acute response as a reduction in mPAP by at least 10 mmHg to reach an absolute value of no more than 40 mmHg, with increased or unchanged cardiac output (23). Nevertheless, it should be interesting to explore the vasoreactivity before cardiac surgery in patients with PH, especially if PVR is high. A vasodilator challenge with inhaled NO during right heart catheterization in assessment of heart transplant candidates is easy, well tolerated and may identify patients at higher risk of RV failure or mortality. For instance, a PVR > 3 WU without reversibility would represent an increased risk for RV failure whereas those with reversibility have the same risk than those with PVR below 3 WU at baseline (56). Preoperative testing of pulmonary vasoreactivity in patients with PH before adult cardiac surgery could predict subsequent positive hemodynamic and perhaps organo-protective responses to inhaled NO and other vasodilators and requires follow up studies.

### 6.2 Effective PVR reduction and translation into clinical benefit

The word “responder” can be applied to curative treatment when some reduction in PVR is observed. However, the threshold of mPAP effective in improving RV function after cardiac surgery should be different than the definition of positive responder in the setting of group 1 PAH (23). This threshold is unknown and should be personalized for each patient and also depends on filling pressures and cardiac output. In a small-sized RCT in mitral valve surgery, postoperative treatment with sildenafil was effective in reducing mPAP but failed to show any difference in hemodynamic parameters and global outcome except for a decrease of in-hospital stay in the sildenafil group vs. placebo (57). Nevertheless, this study did not describe the effect of treatment on organ function, so the authors could not explain this difference. However, it has been reported that residual, but not preoperative, PH was a significant risk factor for postoperative mortality and morbidity (58). In the recent largest observational study on acute PH treatment by inhaled NO, very few cases of treatment discontinuation for lack of efficacy were described, taking into account a large incidence of combined pulmonary vasodilator therapy in the pediatric population but also not negligible in adults (59). Once again, no description of the effect on RV failure and organ function or biomarkers was provided in this study, and these endpoints deserve further exploration. In fact, a positive clinical effect can be achieved when even a slight decrease in RV afterload leads to improved RV function and cardiac output. A global positive clinical outcome can be assessed by the reduction of inotropes and vasopressors use (in dose and duration), the need for mechanical circulatory or RV support, and end organ function preservation (especially for the renal and liver function) by reduction of venous congestion. The evolution of biomarkers associated with RV overload (NT-proBNP or others) should also reflect the positive effect of the treatment on RV distension.

Assessing the effect of preventive use of inhaled NO is even more complex. For prophylactic use, the expected effect is to avoid a deleterious slight increase of pulmonary pressure impairing RV function or rise in another biomarker associated with RV overload. This is current practice but with very low supportive evidence. The main problem to assess the effect of a prophylactic indication is the potential high number of patients to treat, for only a few who will benefit from this strategy. However, the severity and the consequences of acute RV failure following CPB justify the interest in preventing any increase in PVR in high-risk patients (left heart disease with PH undergoing cardiac surgery, i.e., mitral valve disease, LVAD, heart transplantation). The impact of PH on worse outcome after cardiac surgery in adults depends much more on how this PH is managed to avoid RV failure than on the absolute level of PH *per se*. Suggested endpoints for future studies should include the incidence of the low cardiac output syndrome due to RV failure and the incidence of AKI or the highest postoperative Sepsis Related Organ Failure Assessment (SOFA) score.

### 6.3 The clinical redox biology and impaired signaling of inhaled NO

The scientific basis, clinical utility, and potential limitations of inhaled NO largely relate to its free radical nature. It determines the activation of sGC but also makes the NO molecule susceptible to consumption reactions by multiple mediators, especially in an inflammatory environment of increased oxidative stress and free radical generation. These reactions result in marked NO/redox disequilibrium and impaired cardiovascular cell function secondary to oxidative protein modifications. They may render inhaled NO therapy less effective by reducing bioavailability and bioactivity with subsequently impaired cGMP-dependent vasodilatory effects. Notably, the toxic reaction with superoxide to generate peroxynitrite counteracts beneficial effects, including pulmonary vasodilation and organ protection. Of note, assessment of oxidative protein modification and measurements of peroxynitrite and other free radicals cannot be routinely performed in clinical laboratories as they require advanced, highly specialized analytics, including chemiluminescence and electron paramagnetic resonance (EPR) spectroscopy. In addition, indiscriminate use of antioxidants has generally proven unsuccessful in clinical trials, emphasizing the need to understand better the specific sources and spatiotemporal dynamics of toxic radical generation in high-risk patients undergoing cardiac surgery. Better knowledge of inhaled NO-based redox reactions in the individual patient is required to maximize the biological, physiological, and therapeutic effect of inhaled NO. Similarly, few studies have consistently measured circulating cGMP serum levels during inhalation of NO in relation to the presence, extent, or absence of hemodynamic changes, a prerequisite to better understand if and how inhaled NO affects cardiopulmonary homeostasis and pulmonary vascular resistance during CPB. Follow up studies should therefore include targeted measurements of redox biomarkers and circulating cGMP levels.

### 6.4 Systemic inflammatory response to cardiac surgery and influence of inhaled NO

The “systemic inflammatory response” to cardiac surgery remains a popular concept of postoperative complications based on a putative exaggerated innate immune response of the body to surgical trauma, procedure-dependent ischemia-reperfusion injury and cardiopulmonary bypass (60). However, despite many decades of research, the basic pathogenic mechanisms and definitions (61) and the links between inflammatory burden and postoperative single or multiple organ dysfunction remain incompletely understood as evidenced by the failure of anti-inflammatory therapies (62). Adequate assessment of the individual sensitivity, predisposition and responses to surgical and procedural trauma should be prioritized along with the unmet need for early identification, and innovative targeting of confirmed inflammatory phenotypes with personalized therapies.

Because inhaled NO during and following cardiac surgery and CPB may significantly interact with hematological and vascular inflammatory mediators, specific care is needed to avoid formation of injurious toxic intermediaries, e.g., in older patients with significant comorbidities. This population is notoriously prone to a heightened systemic inflammatory response in the perioperative setting and faces increased mortality and hospital length of stay following cardiac surgery as demonstrated in a 10,000 patient large cohort at Cleveland Clinic (63). Unfortunately, most of the available data on the dynamics of endogenous NO generation during cardiac surgery come from preclinical studies, reporting initial overproduction of endogenous NO upon surgical stimulus and CPB caused by eNOS activation, and reduced NO availability at later stages of surgery as part of global endothelial dysfunction. This appears to coincide with induction of iNOS in the myocardium, pulmonary and systemic vascular beds potentially leading to overproduction of NO with subsequent rise in plasma nitrate, nitrite and peroxynitrite concentrations and increased nitrosative stress (64). In cardiac patients, the aggregate impact of inflammatory phenotypes on the balance between NO-mediated cytoprotection vs. cellular, humoral, and metabolic injury remains largely unknown (65) but is a major focus for new clinical translational studies in high-risk populations.

### 6.5 Role of senescence pathways in cardiac outcomes and influence of inhaled NO

It has been well established that more than 95% of the variability in clinical outcomes following cardiac surgery is attributable to age and chronic conditions at baseline, the latter of which increases the likelihood of organ injury and dysfunction via incompletely defined mechanisms. Recent preclinical data indicate that increased baseline cellular senescence with enhanced redox stress and impaired oxidative metabolism increases the susceptibility to acute myocardial and kidney injury. In contrast, reductions in levels of cellular senescence protect kidneys (66–68) and hearts with improved LV remodeling after acute myocardial injury (69, 70). The senescence-related oxidative stress and secretion of so-called SASP, senescence-associated secretory phenotype, creates a pro-inflammatory toxic milieu for neighboring parenchymal cells in the heart and kidneys, resulting in DNA damage, mitochondrial dysfunction, and persistent basal inflammation. Such state of hyperinflammation in patients with multimorbid conditions is referred to as inflammageing and renders them extremely vulnerable to the additional stress of cardiac surgery. It also contributes to hyperlactatemia, a well-recognized clinical predictor of post-surgery organ dysfunction. The interactions between inhaled NO and senescence-related enhanced redox stress markers should be addressed in future studies.

## 7 Barriers to clinical trials

Most of the recommendations and clinical uses that are widely accepted by the scientific community are based on the selective pulmonary vasodilator capacity of inhaled NO. However, the latter effect has not translated into a statistically significant mortality benefit. Such mismatch is not a unique observation exclusively in the setting of inhaled NO but has been repeatedly observed in critical care RCTs because of small sized and heterogeneous target populations. Different surgical scenarios (LVAD, cardiac surgery, lung transplantation, cardiac transplantation) and populations have been recruited (adult, pediatrics). Clinical trials also reported variable definitions of the main outcome (RV dysfunction), variable dosing schedules, and follow-up time periods, and they were generally underpowered for mortality. These flaws in RCT design are responsible for the lack of significance in hard clinical endpoints but, at the same time, suggested the futility of other outcomes different from mortality. The neutral effects on mortality resulting from underpowered RCT may lead to ill-considered refusal of potentially valuable therapies. Moreover, high mortality syndromes, including patients with RV dysfunction, represent a hurdle to conducting genuine RCTs because of crossover between groups as very sick patients present ethical dilemmas to withhold potentially beneficial therapies (71, 72). This situation was also reported in other RCTs with ECMO in ARDS (73).

The discrepancies between clinical and statistically significant effects have also been described in other trials based on the revered *p*-value (74), which expresses a probability of obtaining a result equal to or more extreme than what was actually observed. Alternative approaches, such as Bayesian statistics or high-volume registries, may provide a more intuitive way to interpret data, express uncertainty, and reduce the expenses of RCTs.The scientific community should carefully interpret RCT evidence instead of solely relying on mortality *p*-values and consider the broader context of the inhaled NO effect, understanding the practical implications.

## 8 Barriers for cost effective inhaled NO therapy

The cost-effectiveness of inhaled NO has been the basis of numerous cost-per-quality-adjusted life year (QALY) studies. Lack of statistical significance and cost concerns have limited the use of inhaled NO, ignoring relevant arguments of a treatment with beneficial effects:

- The costs of inhaled NO in the U.S. have been extrapolated to Europe. The costs of medical and surgical treatments are significantly different, motivated, among other factors, by different healthcare systems (75, 76).
- Beneficial effects of inhaled NO have been overlooked, e.g., days on mechanical ventilation (38, 77) and ICU length of stay (39, 78), which could have outweighed inhaled NO costs. Cost-effectiveness analyses should, therefore, also focus on medium- and long-term outcomes that can be particularly relevant in the critical care environment.
- The proposed alternative treatments have not been shown to improve mortality; moreover, the largest prospective randomized blinded study from Ghadimi and co-workers demonstrated a higher rate of death at the different time intervals up to 1 year in participants treated with an alternative vasodilator compared to inhaled NO, but the difference was not statistically significant because of limited sample size and various fatal manifestations of underlying critical illness (79).

In summary, while the medical community at large should prioritize health system sustainability and resource management politics, decision making should be essentially based on the correct interpretation of clinical trials, center expertise, and scientific quality search.

## 9 Opportunities: optimization of inhaled NO therapy in adult and pediatric cardiac surgery

To standardize the use of inhaled NO in cardiac surgery, the following questions need to be answered: what is a reasonable duration of treatment? What is the optimal NO dose? How should the inhaled NO therapy be monitored? What could be a structured weaning protocol to terminate the inhaled NO therapy safely?

### 9.1 What is the optimal timing and reasonable duration of inhaled NO treatment?

When inhaled NO is used in cardiac surgery patients, it is mainly utilized during the perioperative setup. Several studies reported a duration of NO treatment including the time of surgery and up to 24 h in the ICU (3). However, low-dose administration of inhaled NO can be extended to several days and even weeks without any significant side effects (80). In children with congenital heart defects, long term use may be needed (i.e., days to weeks) (81). To determine the optimal duration of inhaled NO administration in the setting of cardiac surgery, targeted phase II studies with variable treatment duration in carefully selected and homogeneous patient cohorts need to be conducted. Appropriate metrics and advanced statistical methodologies to best gauge efficacy need to be carefully considered. We need to integrate the best available surrogate composite endpoints, including MODS scores, biomarkers of myocardial and tubular damage, and cardiopulmonary hemodynamic parameters, and consider the innovative statistical concept of the win ratio for reporting composite endpoints (82).

### 9.2 What is the optimal inhaled NO dose?

Numerous studies evaluated inhaled NO at various concentrations up to 80 ppm, whereas NO doses between 5 and 40 ppm were mostly utilized (37, 38, 83–89). These investigators observed that continuous NO breathing lowers pulmonary arterial pressure and PVR in a dose-dependent manner in the presence of pulmonary vasoconstriction. Intriguingly, doses below 80 ppm do not cause systemic hypotension as NO is rapidly scavenged by intracellular hemoglobin in circulating red blood cells once the drug reaches the pulmonary bloodstream (27). In 2005, a European expert council recommended that NO doses of 20 ppm or lower should be utilized in cardiac surgery patients, as doses greater than 20 ppm failed to show incremental benefit (4, 90, 91).

In neonates and near-term neonates, the recommended dose for the treatment of PH associated respiratory failure is 20 ppm. To minimize the potential adverse effects, the minimal effective dose should be used. Often, doses lower than 5 ppm are effective (92).

### 9.3 How should inhaled NO therapy be monitored?

NO gas readily reacts with oxygen to form higher dioxides, mainly nitrogen dioxide (NO_2_), a pulmonary irritant that may further react to nitric acid in the presence of water-containing fluids (5). To minimize the formation of higher dioxides, medical-grade NO is often balanced in nitrogen, an inert gas, during storage in the gas cylinder. When inhaled NO is delivered, special gas delivery equipment is needed to minimize the duration of exposure to oxygen. Careful and continuous monitoring of both NO and NO_2_ levels is mandatory as the vasodilating effects in the pulmonary vasculature are transient when the delivery is discontinued (2, 4). Inhaled NO providers usually include inhaled NO delivery specific devices that include NO and NO_2_ monitoring with delivery alarms. Frequent measurements of methemoglobin are strongly recommended in particularly when high doses of inhaled NO are administered (2, 4, 27). Doses below 20 ppm significantly reduce inhaled NO-related related side-effects such as formation of methemoglobin and nitric dioxide (4). However, administration of inhaled NO up to 80 ppm for several hours in acute myocardial infarction patients was safe and not associated with increased levels of Met-Hb. In children administered with inhaled NO for days to weeks at the dose of 5–40 ppm, the maximum methemoglobin levels were ≤4% (81). Of note, other aerosolized vasodilators like epoprostenol also need complex delivery and airway system (heat exchanger filter) surveillance in order to prevent delivery abrupt stop and sticking of the particles to the filters and or expiratory valves (79).

Inhaled NO-induced enhancement of RV performance is mediated through different pathways impacting on RV systolic function, RV afterload, and venous congestion. Therefore, cardiac imaging studies of inhaled NO response should include a multi-faceted panel of parameters. However, in the postoperative setting, grading and time trend description of RV systolic dysfunction severity can be challenging because of impossible acoustic windows and the proper RV morphology. Speckle tracking and 3D echo are emergent and promising tools to accurately measure RV function, although time-consuming, impractical, and challenging in the perioperative scenario.

Hence, perioperative assessment of RV function is usually limited to qualitative RV systolic function description and chamber dimension quantification. Longitudinal impairment [tricuspid annular plane systolic excursion (TAPSE)] occurs upon pericardial opening in the absence of clinically significant RV dysfunction and transversal function [fractional area change (FAC)] is known to be preload and afterload dependent. Several echocardiographic studies have failed to demonstrate enhanced RV performance after inhaled NO. Still, limited accuracy and reproducibility preclude the use of RV echocardiographic analysis as an isolated and exclusive parameter. Furthermore, RV systolic function may not be the unique parameter as it may remain unchanged even if inhaled NO response is present by RV afterload reduction.

Doppler echocardiography has also been used to estimate pulmonary pressures and response after inhaled NO. Even if afterload reduction by echocardiography may be a secondary method compared to Swan-Ganz catheter, regression of end systolic interventricular septum flattening and reduced tricuspid regurgitation may indicate better ventriculo-arterial coupling.

Nevertheless, dysfunction and congestion of venous territory that occurs at renal, hepatic, and cerebral areas are the main drivers for MODS and morbimortality in patients undergoing cardiac surgery. Increased central venous pressure may result from systolic and/or diastolic myocardial dysfunction, pulmonary hypertension, and increased intrathoracic pressure. Currently, ultrasound parameters such as portal pulsatility, suprahepatic and renal venous flow patterns, and inferior, superior vena cava or jugular and femoral vein have been reported to be useful in cardiac surgery both for diagnosis of venous congestion and monitoring of diuretic and pulmonary vasodilator responses (93–96). These are fast, easy, and reproducible parameters to correlate with RV echocardiographic function evaluation and to monitor trends before and after initiation of pulmonary vasodilators (including inhaled NO), diuretics and/or inotropes. Taken together, they are well positioned to guide and inform the clinician on inhaled NO response patterns of individual patients.

### 9.4 What could be a structured weaning protocol to safely terminate the inhaled NO therapy?

Rapid withdrawal of inhaled NO can cause rebound PH because of the inhaled NO-induced downregulation of endogenous NO production and increased synthesis of endothelin-1, a strong vasoconstrictor (97–99). Rebound PH and desaturation may be prevented by a slow decrease in the inhaled NO dose up to 1 ppm, with a contextual increase in the FiO_2_. If compatible with lung protective ventilation, prevention of hypercapnia is advisable (100). Imbrication with oral or intravenous pulmonary vasodilators (e.g., sildenafil, bosentan, and prostacyclin) can be taken into consideration to facilitate inhaled NO weaning (98, 101, 102). Such indications are common to both the adult and pediatric populations.

### 9.5 Alternative inhaled NO delivery systems besides mechanical ventilation

Traditionally, inhaled NO is mixed into the breathing circuit of a mechanical ventilator and finally delivered through an endotracheal tube or tracheal canula to the patient (103). Recently, there has been growing interest in the administration of inhaled NO in spontaneously breathing patients after extubation (104, 105). In these cases, inhaled NO can either be delivered via a mechanical ventilator using a tight-fitting face mask or by using a high flow nasal canula. Other delivery systems have been invented to generate NO from air by pulse electrical discharge (106) but these recently developed devices are still at the experimental stage and not yet available on the market.

### 9.6 Other pulmonary vasodilators to treat PAH and/or PVR

Although the focus of this review is on inhaled NO as a selective pulmonary vasodilator, alternative strategies for the treatment of PAH and/or PVR should be briefly mentioned. Prostanoid agonists, including iloprost, epoprostenol, treprostinil and prostaglandin E1 can reduce PAH when administered as an aerosol via the respiratory circuit (39). However, epoprostenol has been shown to cause filter obstruction in the respiratory circuit and has been associated with tracheitis or interstitial pneumonia (107–109). Systemic phosphodiesterase type V inhibitors (sildenafil, tadalafil) are approved for the treatment of PAH, but appear to have only a limited effect on PAH caused by other pathologies such as thromboembolism or other pulmonary diseases (110). As this group of drugs is administered intravenously or orally, they are also classified as non-selective pulmonary vasodilators. Milrinone, a phosphodiesterase III inhibitor, can be administered intravenously to act as a non-selective inodilator (111) or nebulized and administered via the respiratory circuit to act as a selective pulmonary vasodilator (112, 113). Another inodilator is levosimendan, which increases the systolic and diastolic function of both ventricles after intravenous administration and modulates various potassium channels in smooth muscle cells, leading to non-selective pulmonary vasodilation (114). Lastly, systemic endothelin antagonists (bosentan, ambrisentan, tezosentan) are another group of effective non-selective pulmonary vasodilators to treat PAH (115). However, in the absence of robust clinical data no evidence-based recommendations can currently be made for the use in cardiac surgery (39).

## 10 Opportunities: biomarkers for identification of target population and definition of response

Classical biomarkers, readily recognizable to clinicians, have focused on elevated N-terminal proB-type natriuretic peptide (NT-proBNP) and high sensitivity troponin for myocardial stress and injury on lower estimated Glomerular Filtration Rate (eGFR) and elevated urinary protein excretion for kidney injury, and standardized levels of MODS as quantified by the SOFA score. More recent insights into pathways of cellular senescence, persistent inflammation, and metabolic dysfunction in high-risk cardiovascular patients will allow us to investigate and develop new biomarkers to predict and track organ dysfunction (and ultimately clinical outcome) in patients undergoing cardiac surgery. They include specific secreted proteins, including soluble urokinase-type plasminogen activator receptor (sUPAR), Interleukin-6, tissue inhibitor of metalloproteinase-2, TIMP-2 and Insulin Growth Factor Binding Protein-7, IGFBP7 as well as metabolites or nucleic acids (that recapitulate the molecular fingerprint of inflammageing and immunosenescence). They can be tracked in circulating blood or in defined populations of white cells (see Supplementary Table 1). In addition, flow cytometry-based measurements of immunosenescence. including naïve to memory T-cell ratios, senescent and exhausted T-cells, and Seahorse XFe24-based measurements of leucocyte mitochondrial function will allow better quantification of the role of innate immunity in organ dysfunction post-cardiac surgery. Finally, the tight link between chronic inflammation, endothelial dysfunction, and platelet activation in high-risk cardiovascular patients undergoing cardiac surgery is reflected in increased levels of circulating prothrombotic and proinflammatory markers (IL-1, hsCRP, fibrinogen, D-dimers, soluble tissue factor VII).

## 11 Recommendations

### 11.1 International consensus for state of the art of inhaled NO therapy

While the current Advisory Board represents a cross-section of clinical leaders of the field, our review is designed to identify persistent barriers and potential avenues for future development. As such, it lacks formal professional society affiliation. The contributors act in their personal capacity and cannot cover the entire preclinical and clinical aspects of the field. This is better achieved by a true collaboration between all stakeholders and a call for concerted action by different clinicians, scientists, and industry to move the field forward. One possibility could be a multisociety consensus conference further debating and employing Delphi methodology to cover all relevant aspects of inhaled NO-based treatment in cardiac surgery, identifying priorities and setting the best way forward. The recent alliance between the inhaled NO-producing company Air Liquide and ESAIC and the establishment of a society-approved Steering Committee and reaching out to EACTAIC could serve as an example to start building a multisociety and industry-clinical alliance. Based on previous and recent collaborations, we envision expanding this collaboration to relevant surgical, cardiology, intensive care, and basic science societies. It will also be important to represent the patient's views and priorities, especially in the high-risk surgical setting.

### 11.2 Improving understanding of real-life effects of inhaled NO therapy in large scale observational studies

While large RCTs provide the highest level of current evidence, the widespread use of inhaled NO therapy worldwide and dedicated clinical leads in these centers provide an invaluable resource for gathering additional up-to-date information on the effectiveness or lack thereof. These observational studies of inhaled NO would facilitate the engagement of larger patient populations than currently achievable with RCTs, especially in the context of current funding limitations.

A good example of this is the recent evaluation of inhaled NO therapy in cardiac surgery in Japan (116). This post marketing evaluation confirmed real-world safety and effectiveness of inhaled NO therapy for PH in *n* = 2,817 patients in Japan (116). In this observational study, inhaled NO treatment was associated with significant reductions in both central venous pressure among pediatric patient and in mPAP among adults and significant improvements in PaO_2_/FiO_2_ in both populations with PaO_2_/FiO_2_ ≤ 200 at baseline.

Our recent surveys also concluded that large observational studies are needed to further evaluate current inhaled NO practices worldwide. Ringfenced funding to specialist societies by pharma industries could serve as a proper framework to facilitate and support such surveys. These observational studies could commence with retrospective analyses that could provide and establish future focus for prospective evaluations in particular surgical conditions and patient populations.

### 11.3 Explaining clinical effectiveness through enhanced biological phenotyping

Our analysis above prompts for much needed clinical biology and chemistry knowledge to capitalize on the beneficial actions of inhaled NO or explain clinical failures. These studies should build on current international efforts to analyze representative biomarkers in blood, urine, or myocardial tissue samples of high-risk patients undergoing cardiac surgery and investigate their relation to post-surgery, myocardial, pulmonary, brain, and kidney dysfunction. As these studies have already identified various aspects of inflammation, metabolic dysfunction, and immune activation with post-surgery organ dysfunction, our next step will be to investigate the effect of inhaled NO on these pathogenic pathways. This can be performed in a first step as a translational pilot accompanying the retrospective observational trials by analyzing available biorepositories. These translational studies could focus on biomarkers discussed above as candidate mediators markers of organ dysfunction, but should be extended to unbiased phenotyping approaches to define the global transcriptomic, proteomic, and metabolic phenotypes related to inhaled NO therapy. If available, biomarker data need to be correlated and integrated with available imaging and hemodynamic parameters, which will help in better understanding responses to inhaled NO in cardiac surgery patients. In a second approach, results should be confirmed in sufficiently powered prospective studies of high-risk cardiovascular patients undergoing cardiac surgery.

### 11.4 Innovative future RCT designs

The above observational and translational studies should provide a clear understanding of the variability and heterogeneity of the clinical, physiological, and molecular responses to inhaled NO in high-risk patient populations and will help identifying the best future targets, populations and design of clinical trials towards effective clinical translation of the current encouraging preclinical data.

Similarly, the translational studies should provide directions regarding the value of the biomarkers, and the preoperative determination of clinical and biological phenotypes that could either improve patient selection and/or better predict treatment response. For instance, understanding the responder-non-responder phenotypes would allow us to enrich high-risk patient groups with focusing on the responder group only thereby maximizing the opportunity for inhaled NO to improve outcomes. Alternatively, this information will inform the design of meaningful trials to convert non-responders (for instance, with sGC resistance) and restore inhaled NO responsiveness.

It is clear that these trials should target high risk populations as the majority of cardiac surgical patients fortunately have an uneventful perioperative journey without organ dysfunction or major morbidity. Accordingly, mortality should not serve as sole primary efficacy outcome in trials of high-risk patients undergoing cardiac surgery. Instead, these clinical trials on the perioperative use of inhaled NO in high-risk cardiovascular patients should consider as the primary objective (or should be primarily directed towards) the prevention of organ injury and dysfunction (to RV and LV but also to the brain, kidneys and lungs in patients undergoing CPB). In these trials, inhaled NO should be given an opportunity and time to influence the critical events and underlying biology of the evolving organ dysfunction with appropriate delivery and optimized dose and duration of treatment. We should also design pilot studies to investigate a putative curative effect of peri-operative inhaled NO on organ injury (often caused by ischemia reperfusion injury) and gauge its clinical effect accordingly.

These trials should be paralleled with appropriate translational studies focusing on the carefully chosen surrogate markers and the global proteomic and metabolic phenotypes to facilitate a better understanding of the pathogenesis of organ dysfunction and the potential therapeutic or prophylactic role of inhaled NO.

It is also likely that these trials will have parallel arms, for instance. to enhance the effectiveness of inhaled NO therapy with complimentary intervention arms. Among these, targeting the NO consumption reactions, anti-oxidants, sGC sensitizers and the potential additive action of inhaled NO with other pulmonary vasodilators will deliver a modern approach to maximize inhaled NO therapy.

## 12 Conclusion

In this review, we discussed why inhaled NO is currently at a crossroads in cardiac surgery. We explained the reader why the improvement of the mechanistic understanding is needed, and how the clinical trial design and the scientific evidence should be improved to identify the persistent barriers and potential avenues to help guiding more robust and informative randomized controlled trials in the future. The efficient conduct of these trials will require a dedicated international research network, multiple support by all relevant industries, society engagement and appropriate clinical trial units experienced with platform clinical trials and adaptive clinical trial design.
