# Supplementary material for: Inhaled NO at a crossroads in cardiac surgery: current need to improve mechanistic understanding, clinical trial design and scientific evidence
Source: Front Cardiovasc Med. 2024 Apr 5;11:1374635. doi: 10.3389/fcvm.2024.1374635 (PMC11027901; doi:10.3389/fcvm.2024.1374635)
Supplement: Supplementary file 1 [file Table1.pdf]

Supplementary Material

1 Supplementary Table 1

Supplemental table 1: Biomarkers related to myocardial and kidney dysfunction in high-risk cardiovascular patients undergoing cardiac surgery and potentially modifiable by inhaled NO

A. *markers of organ dysfunction/injury*:

- *cardiac:*
    - *hs Troponin*
    - *Nt-proBNP*
- *kidney:*
    - *serum creatinine*
    - *eGFR*
    - *Cystatin-C*
    - *Urinary protein excretion*
    - *neutrophil gelatinase-associated lipocalin*
    - *echocardiographic assessment of renal venous congestion*
    - *urinary salt excretion*

B. *markers of (immune)senescence, inflammation, thrombosis, metabolic dysfunction, and inflammageing in chronic disease:*

- *senescence-associated secretory proteins (SASP)*
    - *IGFBP7*
    - *TIMP2*
- *hsCRP, Interleukin-6, Interleukin-1*
- *FACS analysis of senescent T-cells, exhausted T cells,…*
- *serum soluble urokinase-type plasminogen activator receptor (sUPAR)*
- *leukocyte mitochondrial function*
- *leukocyte myeloperoxidase*
- *fibrinogen, D-dimers, and soluble tissue factor VII*

C. *markers of NO signaling and redox state*

- *reactive oxygen species: NO_2-/NO_3-, PgF_2 alpha, nitrotyrosine, peroxynitrite,…*
- *circulation and urinary cGMP (EIA)*
- *MetHb*
- *SNO-Hb, SNO-proteins*
